# Relationship Between Gut Microbiota and the Clinical Course of COVID-19 Disease

**DOI:** 10.3390/v17040520

**Published:** 2025-04-02

**Authors:** Antonija Jonjić, Ivan Dolanc, Goran Slivšek, Luka Bočkor, Marko Tarle, Sanda Mustapić, Marta Kmet, Biserka Orehovec, Paola Kučan Brlić, Maja Cokarić Brdovčak, Ante Obad, Martin Walenta, Ivan Dražić, Lidija Bilić-Zulle, Ivica Lukšić, Neven Bulić, Walter Goessler, Stipan Jonjić, Miran Čoklo, Jurica Žučko

**Affiliations:** 1 Institute for Anthropological Research, 10000 Zagreb, Croatia; ivan.dolanc@inantro.hr (I.D.); goran.slivsek@xnet.hr (G.S.); lbockor@inantro.hr (L.B.); 2 Dubrava University Hospital, 10000 Zagreb, Croatia; tarlemarko1@gmail.com (M.T.); zmarta.km@gmail.com (M.K.); biserka.orehovec@gmail.com (B.O.); luksic@kbd.hr (I.L.); 3 School of Dental Medicine, University of Zagreb, 10000 Zagreb, Croatia; 4 Faculty of Medicine, University of Rijeka, 51000 Rijeka, Croatia; paola.kucan@medri.uniri.hr (P.K.B.); maja.cokaric@medri.uniri.hr (M.C.B.); lidija.bilic.zulle@medri.uniri.hr (L.B.-Z.); stipan.jonjic@medri.uniri.hr (S.J.); 5 University Department of Health Studies, University of Split, 21000 Split, Croatia; aobad7@gmail.com; 6 Institute of Chemistry, Analytical Chemistry, University of Graz, 8010 Graz, Austria; martin.walenta@uni-graz.at (M.W.); walter.goessler@uni-graz.at (W.G.); 7 Faculty of Engineering, University of Rijeka, Vukovarska 58, 51000 Rijeka, Croatia; ivan.drazic@riteh.uniri.hr (I.D.); neven.bulic@riteh.uniri.hr (N.B.); 8 Rijeka University Hospital Centre, 51000 Rijeka, Croatia; 9 School of Medicine, University of Zagreb, 10000 Zagreb, Croatia; 10 Faculty of Food Technology and Biotechnology, University of Zagreb, 10000 Zagreb, Croatia; jzucko@gmail.com

**Keywords:** 16S rRNA analysis, clinical course, COVID-19, gut microbiota, SARS-CoV-2

## Abstract

Possible early detection of people at increased risk for severe COVID-19 clinical course is extremely important so that appropriate therapy can be initiated promptly to prevent numerous deaths. Our study included 45 patients treated for COVID-19 at Dubrava University Hospital, with clinical course analysed from medical records and stool samples collected for determination of the gut microbiota diversity using 16S rRNA analysis. Sequencing was successful for 41 samples belonging to four clinical course groups (WHO guidelines): 12 samples—critical, 12—severe, 9—moderate and 8—mild group. Microbial composition was assessed between groups using two approaches—ANCOM (QIIME2) and Kruskal–Wallis (MicrobiomeAnalyst). On the genus level, two taxa were found to be differentially abundant: archaeal Halococcus and Coprococcus (for both W = 37)—the two were most abundant in the critical group (10% and 0.94% of entire abundance, respectively). Coprococcus catus was the only species identified by both methods to be differentially abundant between groups and was most abundant in the critical group. Alpha diversity indicated greater evenness of features in the critical group. Beta diversity showed clustering of samples from the critical group. A relationship between gut microbiota composition and the clinical course of COVID-19 disease was indicated, pointing towards specific distinct features of the critical group. In a broader sense, our findings might be useful in combating potential future similar pandemics and emerging virus outbreaks.

## 1. Introduction

COVID-19 is an infectious disease caused by a novel coronavirus SARS-CoV-2, which resulted in a catastrophic global crisis [1]. The possibility of early detection of people at increased risk of developing a severe clinical picture is extremely important so that appropriate therapy can be initiated promptly to prevent numerous deaths.

The composition of the gut microbiota plays a critical role in human health and disease, with an emphasis on immunological homeostasis [2]. There are several studies suggesting that gut microbiome is involved in the magnitude of COVID-19 severity, possibly via modulating host immune responses [3]. Today, we know that gut microbiota influences the immune response to lung infections via the gut–lung axis [4]. People with underlying medical conditions (comorbidities) such as diabetes, obesity, and hypertension (which are entities known to be related to specificities in gut microbiota composition) are at a significantly higher risk of developing severe COVID-19 clinical course [5]. Elevated ACE2 receptor expression increases the exposure of type 2 pneumocytes to the SARS-CoV-2 spike protein and is influenced by various factors. The gut microbiota influences ACE2 receptor expression, while ACE2 simultaneously influences the composition of the gut microbiota [6].

The interplay between the SARS-CoV-2 and gut microbiota composition becomes even more complex if we take into account that today we know that SARS-CoV-2 is bacteriophagic and, as such, can, to some extent, directly influence the gut microbiota composition [7,8].

On the other hand, among numerous factors that influence gut microbiota composition, antibiotic use and diet are perhaps the most commonly mentioned [9]. Antibiotic use is generally known to reduce microbial diversity of the gut microbiota, but also to alter its composition in terms of species abundance and to promote growth of the antibiotic-resistant bacteria [10]. Antibiotics often decrease the variety of bacterial species in the gut microbiome, leading to a less diverse microbiome and potentially allowing harmful bacteria (such as Clostridioides difficile) to overgrow [10]. Antibiotic use can reduce or deplete beneficial species, such as Bifidobacterium and butyrate-producing bacteria, which can persist for months after treatment [11]. Finally, antibiotics promote the growth of antibiotic-resistant bacteria by selecting them through the elimination of susceptible strains, increasing the abundance of bacterial resistance genes in the microbiota gene pool [10]. While some microbiotas recover within weeks, some species may remain undetectable for longer periods of time, thus potentially affecting metabolic and immune functions, which is of importance regarding the clinical course of existing diseases [11]. In addition, it is known that critically ill patients show rapid changes in gut microbiota composition in terms of depletion of health-promoting microorganisms [12].

Diet also plays an important role in shaping gut microbiota composition by influencing its bacterial diversity, abundance of certain species, as well as metabolic activity [13]. Western diet (high in fat, protein and sugar, but low in fibre) promotes Bacteroides and bile-tolerant bacteria, at the same time reducing beneficial species like Firmicutes and Bifidobacteria [13]. On the other hand, plant-based diets (rich in fibre), including the Mediterranean diet (rich in fibre, polyphenols and healthy fats), increase the abundance of beneficial bacteria like Prevotella, butyrate-producing and short-chain fatty acid-producing species, at the same time boosting bacterial diversity [13].

Our study, therefore, aims to investigate the relationship between gut microbiota composition specificities and the severity of the clinical course of COVID-19 disease.

## 2. Materials and Methods

### 2.1. Patients

Our study included 45 COVID-19 patients treated from June 2021 to December 2021 at the Dubrava University Hospital, Zagreb, Croatia. Single fix time-point study design was used. For 4 patients, DNA sequencing was unsuccessful, while for the rest of 41 patients, clinical course of COVID-19 disease was analysed from medical records and stool samples were collected following admission to hospital for determination of the diversity of gut microbiota in stool samples using 16S rRNA analysis. Sequencing resulted in 41 sets of demultiplexed paired-end fastq files. Out of 41 patients aged between ages 24 and 95 (median 65), there were 20 males and 21 females.

### 2.2. Clinical Classification

Patients with COVID-19 were classified into four groups based on the WHO ordinal scale for COVID-19 Disease Severity Classification [14].

The groups were as follows: (1) Mild—Symptomatic patients meeting the case definition for COVID-19 without evidence of viral pneumonia or hypoxia; (2) Moderate—Pneumonia, with SpO2 ≥ 90% on room air; (3) Severe—Severe pneumonia (fever, cough, dyspnoea) plus one of the following: respiratory rate > 30 breaths/min; severe respiratory distress; or SpO2 < 90% on room air; (4) Critical—Worsening of respiratory symptoms, with ARDS, sepsis, septic shock, acute thrombosis.

A table with the relevant symptoms (according to the WHO classification) and duration of illness of the 41 patients is included as Supplementary Data (Table S1).

### 2.3. Sample Preparation and 16S rRNA Gene Sequencing

Stool samples were collected in a manner that they contained both stool’s outer surface and core. They were immediately homogenised and stored at −80 °C. Transportation was performed in dry ice at −70 °C, and samples were again stored at −80 °C. Before the DNA isolation, stool samples were stabilised at 4 °C.

DNA was isolated from stool samples using the QIAamp PowerFecal kit (Qiagen, Hilden, Germany), according to the manufacturer’s protocol and quantified fluorometrically on the Qubit version 3.0 (Invitrogen, Eugene, OR, USA) using the Qubit dsDNA high sensitivity assay kit (Life Technologies, Carlsbad CA, USA). The V3-V4 region of the bacterial 16S rRNA gene was amplified using the primer set 341F-806R with sequence of the primers PRK341CCTAYGGGRBGCAACAG and PRK806RGGACTACNNGGGTATCTAAT. Pooled libraries were sequenced using the Illumina MiSeq platform, following paired-end sequencing protocol (MiSeq v3 Reagent Kit) (Illumina, Inc., San Diego, CA, USA) in Molecular Research (MRDNA) Lab, TX, USA.

Gut microbiome data sequenced in this study are available from EBI’s ENA repository with study accession id: PRJEB81810.

### 2.4. Bioinformatic Analyses

Analysis of amplicon data was conducted using “Quantitative Insights into Microbial Ecology 2” (QIIME2) software [15], release 2022.2. Raw, demultiplexed paired-end fastq files were imported into QIIME2 using the manifest file. Imported sequences were denoised, dereplicated and filtered for chimaeras using the DADA2 plugin (q2-dada2) [16]. Forward reads were trimmed for 17 bases and truncated at position 290, while reverse reads were trimmed for 21 bases and truncated at position 270. Resulting amplicon sequence variants (ASVs) were aligned with mafft [17] using q2-alignment plugin and were used to construct a phylogenetic tree using fasttree2 [18] via the q2-phylogeny plugin. Alpha (observed features, Shannon’s diversity, Pielou’s evenness index and Faith’s phylogenetic diversity) and beta diversity (weighted and unweighted Unifrac, Jaccard distance and Bray Curtis dissimilarity) metrics and principal coordinate analysis (PCoA) were calculated using QIIME2 q2-diversity plugin on datasets rarefied to 30 000 sequences per sample. Taxonomy was assigned to ASVs using a pre-trained Naïve Bayes classifier [19]. As a referent taxonomic database, full-length GSR database [20] was used. Downloaded full GSR database was used to train the classifier on sequences corresponding to variable regions 3 and 4 of the 16S rRNA gene using the primers 341F and 806R used for the sequencing, with the default cut-off value (0.7) used for taxonomy assignment. Beta diversity analysis was carried out using principal coordinates analysis (PcoA) using diversity matrices of all beta diversity metrics, and difference in groups’ microbial composition was assessed via q2-diversity plugin using PERMANOVA test statistics pseudo-F with 999 permutations and q-value of 0.05 considered as significant. Calculated alpha diversity metrics were used to assess significant differences between groups using Kruskal–Wallis test with Benjamini–Hochberg procedure used for false discovery rate via q2-diversity plugin.

Differential abundance between groups, defined based on WHO classification, was obtained using ANCOM [21] via q2-composition plugin on collapsed feature table on all taxonomic levels. Differential abundance using MicrobiomeAnalyst platform was calculated using Kruskal–Wallis test on exported ASV table using *p*-value of 0.05 as significant and with Benjamini–Hochberg false discovery rate (FDR) correction.

Taxonomic abundance visualisations were produced by MicrobiomeAnalyst platform based on exported ASV table and taxonomy. Features present in less than 4 copies and in less than 10% of samples were removed for visualisation while all calculations (differential abundance, alpha and beta diversity) were completed on original unfiltered data.

## 3. Results

### 3.1. Obtained Sequencing Depth

**Figure 1:**
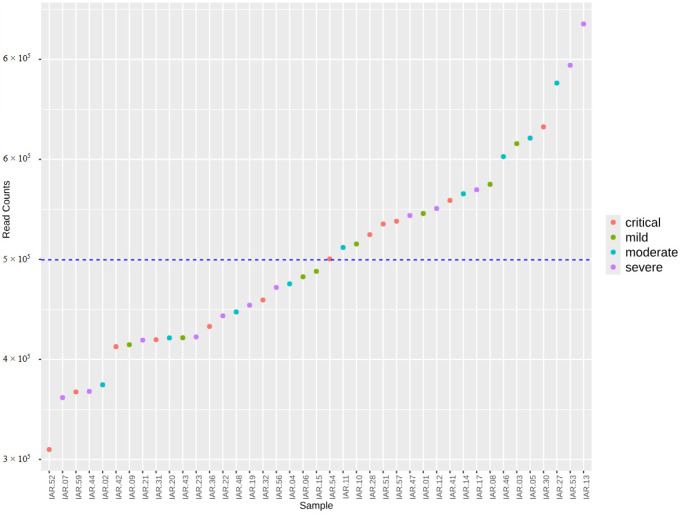
Read counts per sample after quality control filtering by DADA2 algorithm.

Sequencing resulted in 41 sets of demultiplexed paired-end fastq files. Read counts per sample ranged from 58,788 to 133,022, with the average read count per sample being 93,411 ± 17,033 before cleaning and filtering. After quality control and chimaera filtering, read counts ranged from 73,541 to 31,193, with the average depth per sample being 50,494 ± 9549 (Figure 1). No significant differences in sequencing depth between WHO groups were found in either the raw or cleaned data. In total, 2250 amplicon sequence variants (ASVs) were detected in the dataset.

### 3.2. Taxonomic Abundance

In total, 41 samples belonged to four groups based on the WHO guidelines for COVID-19 clinical management: 12 samples in critical, 12 in severe, 9 in moderate and 8 in mild group.

**Figure 2:**
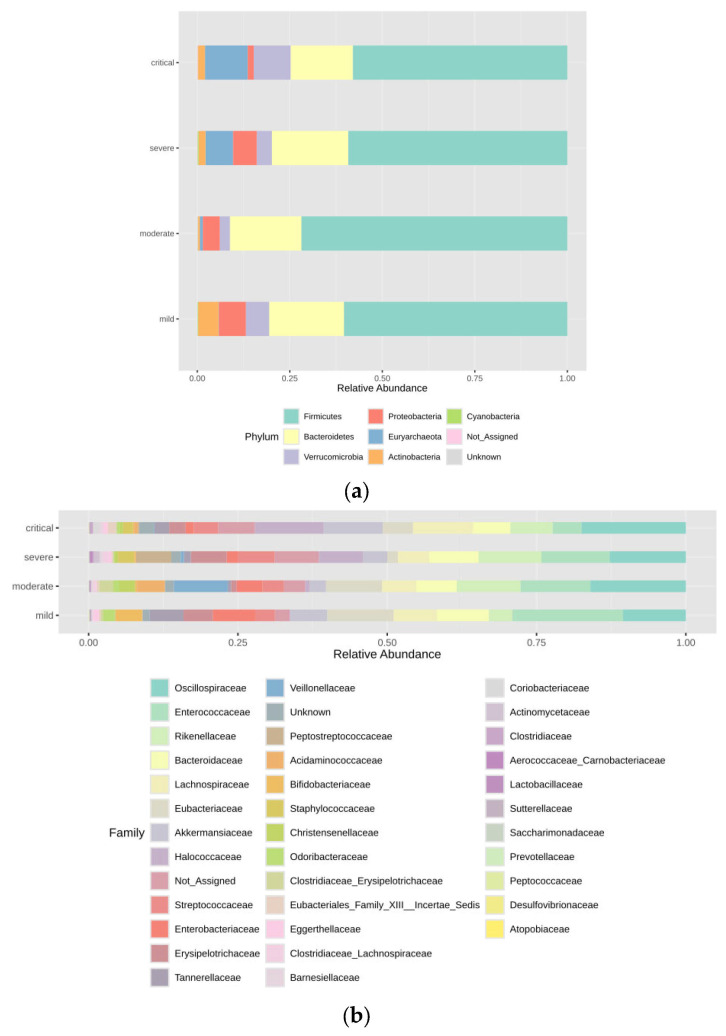
(**a**) Taxonomic abundance between groups at the phylum level. (**b**) Taxonomic abundance between groups at the family level.

The taxonomic composition between groups was fairly similar, being dominated at the phylum level (Figure 2a) by Firmicutes (55.87–67.35%) and Bacteroidetes (19.33–20.92%). The third most abundant phylum was Proteobacteria (6.42–7.2%) in mild, moderate and severe groups, while in the critical group, Archaeal phylum Euryarchaeota was the third most dominant, with frequency of 10.28%. At the family level (Figure 2b), 5 bacterial families made up to 50% of all the abundance—Oscillospiraceae (10.49–17.57%), Enterococcaceae (18.6–4.78%), Rikenellaceae (3.92–10.67%), Bacteroidaceae (6.94–8.67%), Lachnospiraceae (5.18–9.65%) and Eubacteriaceae (4.98–11.14%). Interestingly, the family Halococcaceae was present in severe (7.45%) and critical (11.40%) groups, but not present in the mild group with an abundance of 0.69% in the moderate group.

Concerning the taxonomic assignment, confidence values for the assignment of ASVs were all above the threshold (0.7) but had a “mid-level” confidence score (0.79–0.87) (Figure S1). Confidence values are the raw probability estimates output by the naive Bayes classifier and as such do not give the level of reliability of assignment on different taxonomic levels, as is the case with similarity-based methods. A BLAST (version 2.16.1) search of several representative sequences of ASV assigned to Halococcus returned mostly uncultured Halococcus bacteria with an e-value of e-44, with the identified Halococcus thailandensis not showing in the first half of the results. Results indicate correct taxonomic assignments on higher taxonomic levels—genus Halococcus or family Halococaccaceae.

**Figure 3:**
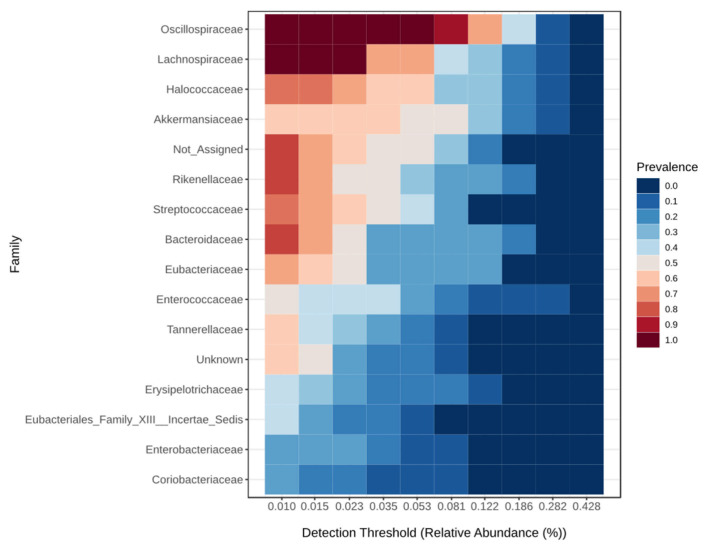
Core microbiome at the family level for critical group.

**Figure 4:**
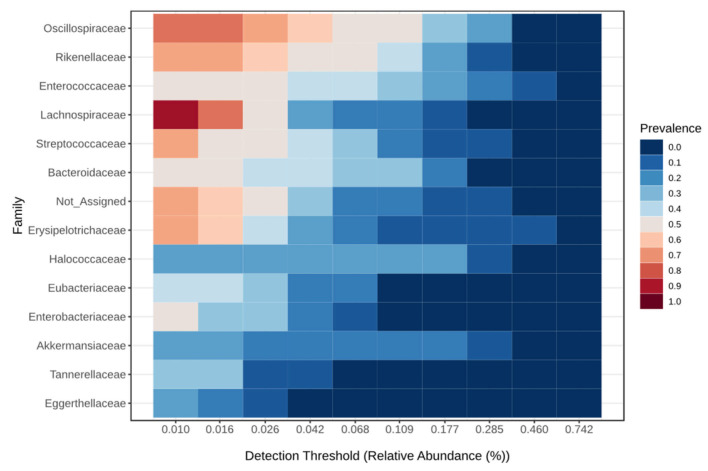
Core microbiome at the family level for severe group.

**Figure 5:**
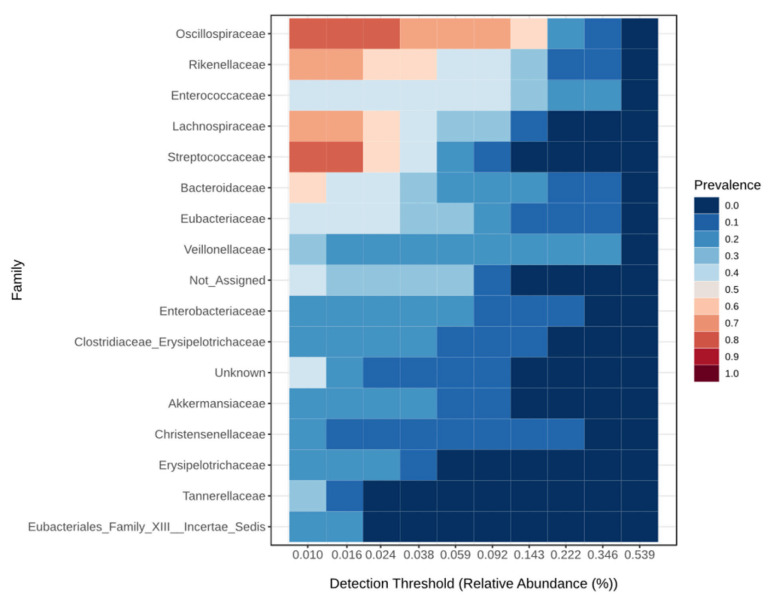
Core microbiome at the family level for moderate group.

**Figure 6:**
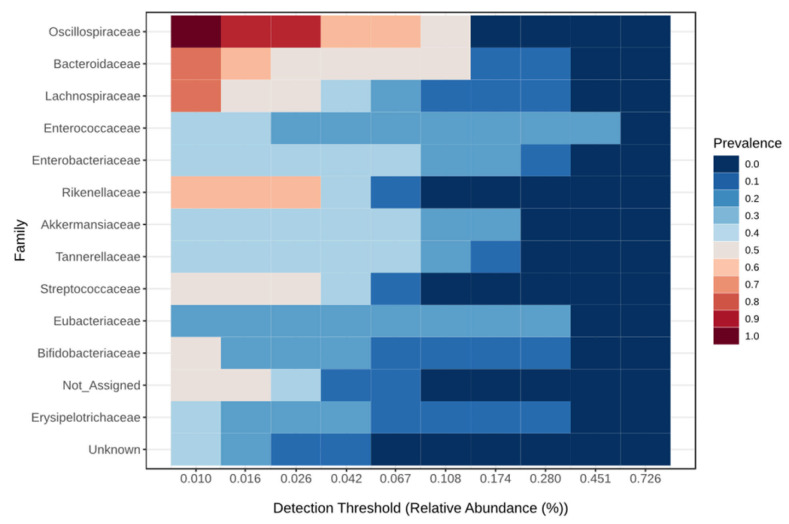
Core microbiome at the family level for mild group.

The core microbiome at the family level for the critical group is shown in Figure 3, for the severe group in Figure 4, for the moderate group in Figure 5, and for the mild group in Figure 6. On the species level 6, the most abundant species make up to 30% of all abundance, those are unidentified bacteria from the genus Enterococcus, Eubacterium coprostanoligenes, Akkermansia muciniphila, Halococcus thailandensis, Alistipes onderdonkii and Faecalibacterium prausnitzii. Interesting is the appearance of Halococcus thailandensis in critical and severe groups where it amounts to 10.28 and 6.78% of all microorganisms.

### 3.3. Differential Abundance

Microbial composition was assessed for differentially abundant taxa between groups, defined based on WHO classification, using two approaches—ANCOM, as implemented in QIIME2 and Kruskal–Wallis test implemented in MicrobiomeAnalyst.

#### 3.3.1. ANCOM Results

The microbiota composition table was collapsed on each of the taxonomic levels, which were then analysed using ANCOM. Only one feature up to the family level was found to be significantly differentially abundant between groups—family Halococcaceae order Halobacteriales, class Halobacteria, phylum Euryarchaeota of the kingdom Archaea, with W value ranging from 16 at the phylum level to 68 at the family level (Table 1). On the genus level, two taxa were found to be differentially abundant, the previously identified archaeal genus Halococcus and bacterial genus Coprococcus, both having a W value of 37. Halococcus was most abundant in the critical group, accounting for more than 10% of the entire abundance, less abundant in severe (7.45%) and moderate (0.79%) groups, while almost not present in the mild group. The other differentially abundant genus Coprococcus, does not show decreasing abundance with the easing of the severity of symptoms, being most abundant in the critical group (0.94%) and followed by the moderate (0.3%) group. On the species level, only one feature, Coprococcus catus, was determined significant, with a W value of 183.

#### 3.3.2. Kruskal–Wallis Results

**Figure 7:**
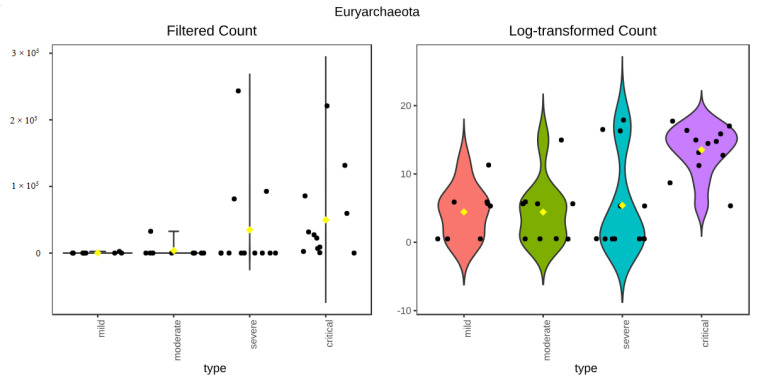
Differentially abundant phylum identified by Kruskal–Wallis test.

**Figure 8:**
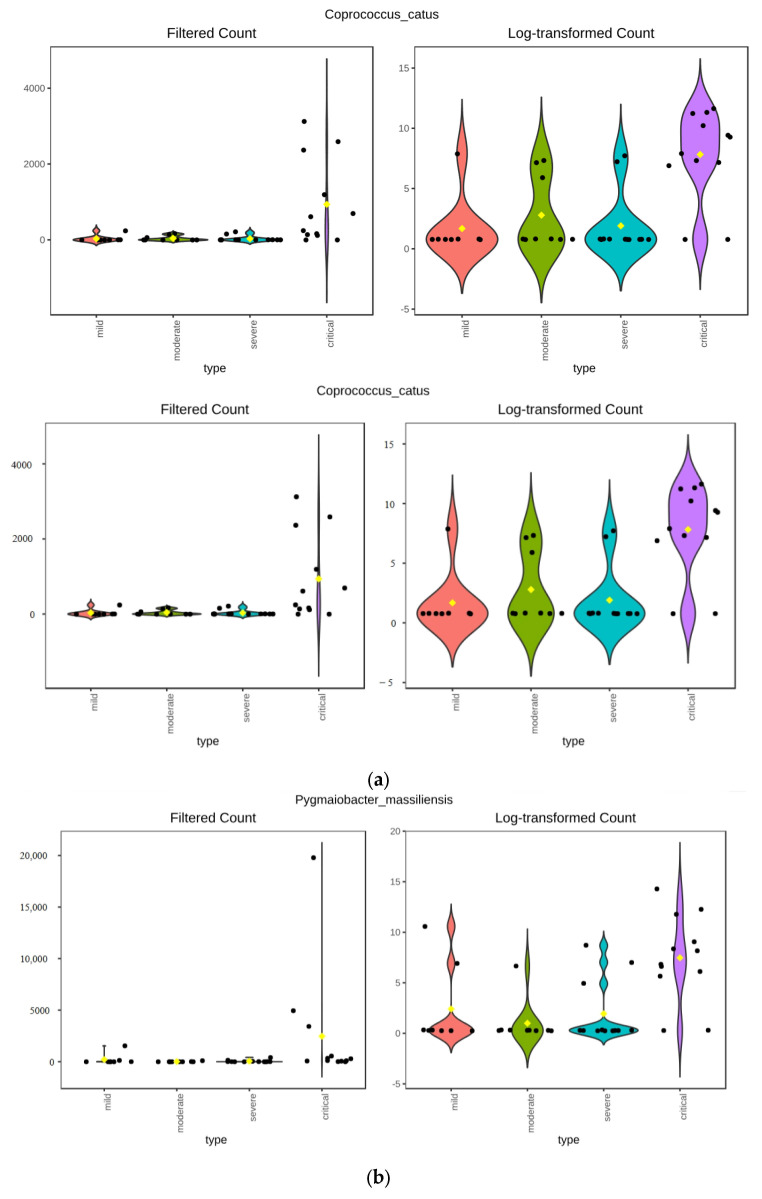

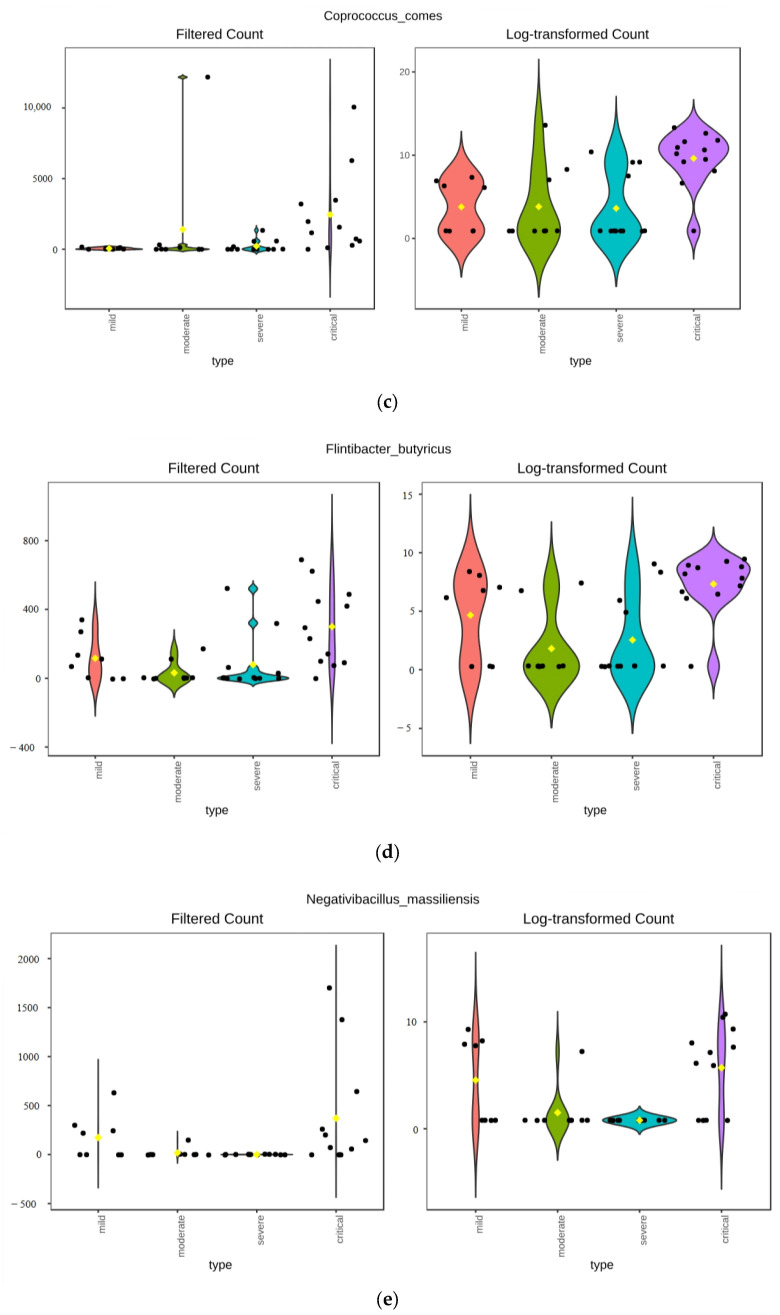
(**a**) Differentially abundant species Coprococcus catus identified by Kruskal–Wallis test. (**b**) Differentially abundant species Pygmaiobacter massiliensis identified by Kruskal–Wallis test. (**c**) Differentially abundant species Coprococcus comes identified by Kruskal–Wallis test. (**d**) Differentially abundant species Flintibacter butyricus identified by Kruskal–Wallis test. (**e**) Differentially abundant species Negativibacillus massiliensis identified by Kruskal–Wallis test.

Analysis of differential abundance based on the Kruskal–Wallis test, as implemented in the MicrobiomeAnalyst platform, showed similar results—on phylum, class and order level, differentially abundant features were the same as identified by ANCOM (Figure 7). Differences arose on the family level, where the Kruskal–Wallis test did not detect Holococcaceae as a differentially abundant feature. On the genus level, the Kruskal–Wallis test identified more differentially abundant features than ANCOM, with genera Coprococcus and Halococcus identified by both. Genera Pygmaiobacter, Flintibacter and Negativibacillus were identified only by the Kruskal–Wallis test as differentially abundant between groups. Species-level analysis identified five species as differentially abundant—Coprococcus catus, Pygmaiobacter massiliensis, Flintibacter butyricus, Coprococcus comes, and Negativibacillus massiliensis (Figure 8a–e). Coprococcus catus was the only feature on the species level identified by both methods to be differentially abundant between groups and most abundant in the critical group.

### 3.4. Alpha Diversity

**Figure 9:**
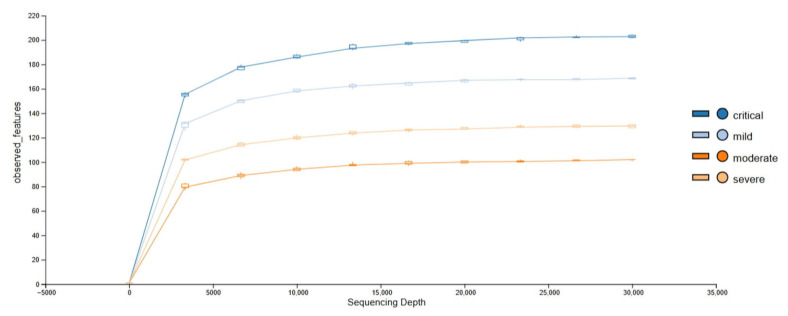
Alpha rarefaction curve for observed features metric.

**Figure 10:**
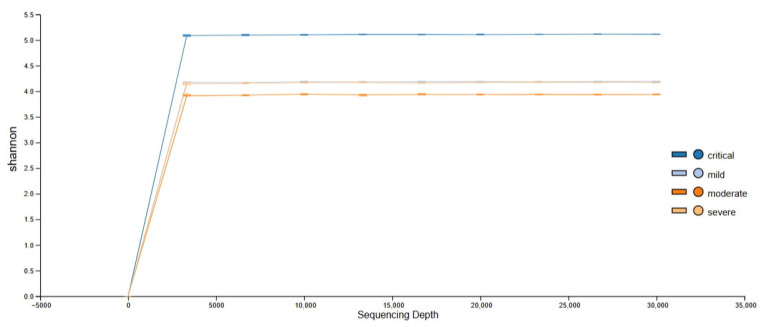
Alpha rarefaction curve for Shannon index metric.

**Figure 11:**
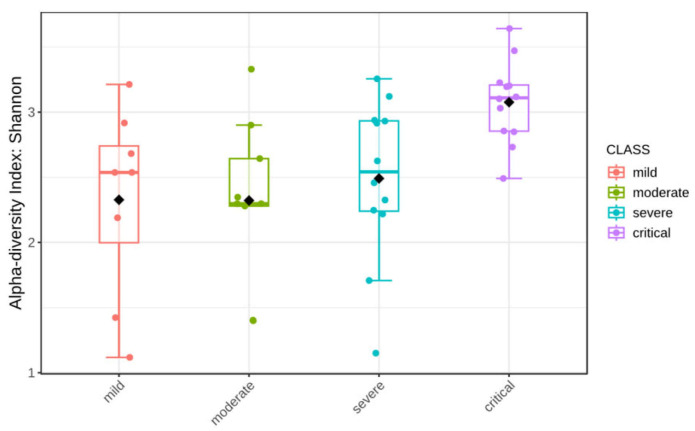
Alpha diversity Shannon index (*p*-value: 0.011996; Kruskal–Wallis statistic: 10.951).

**Figure 12:**
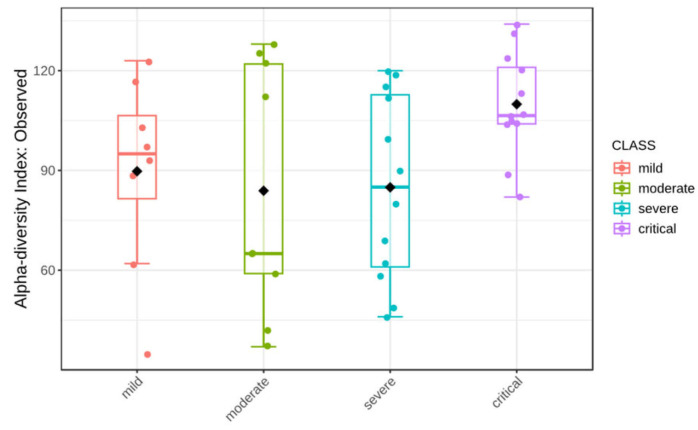
Alpha diversity observed index (*p*-value: 0.1585; Kruskal–Wallis statistic: 5.1886).

Rarefaction curves (based on a sample with the lowest number of reads) show that sequencing depth was sufficient to fully account for the diversity of all sequenced samples (Figure 9 and Figure 10). Calculated were Shannon’s diversity, evenness, and observed features indices that were tested for significant differences between groups, using the Kruskal–Wallis test implemented in QIIME2 and MicrobiomeAnalyst platform. Although the observed feature index was highest for the critical group, it did not show statistically significant differences between groups, while Shannon and evenness indices showed significant differences between critical–mild, critical–moderate and critical–severe groups, indicating greater evenness of the features in the critical group (Figure 11 and Figure 12). Raw data from sequencing did not show significant differences between groups (Table S2), and after cleaning the data (DADA2), no significant differences between groups were found (Table S3). Multiple testing adjustment was completed using the Benjamini–Hochberg procedure (FDR).

### 3.5. Beta Diversity

**Figure 13:**
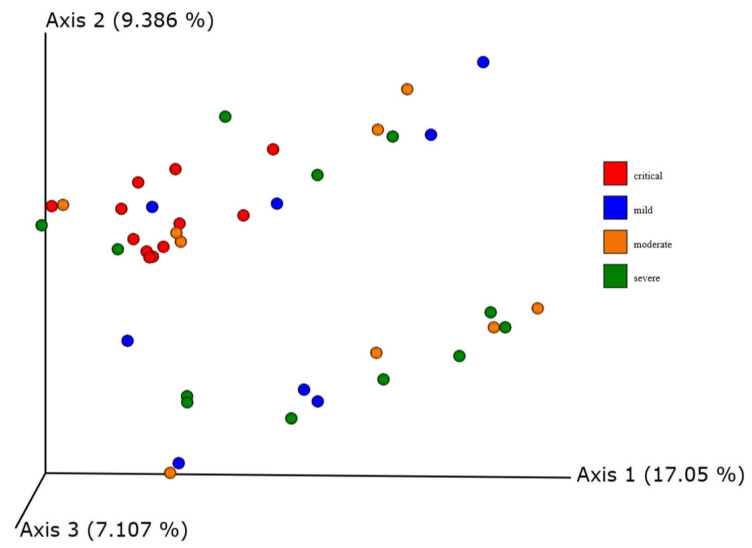
Beta diversity unweighted UniFrac distances—clustering of samples.

**Figure 14:**
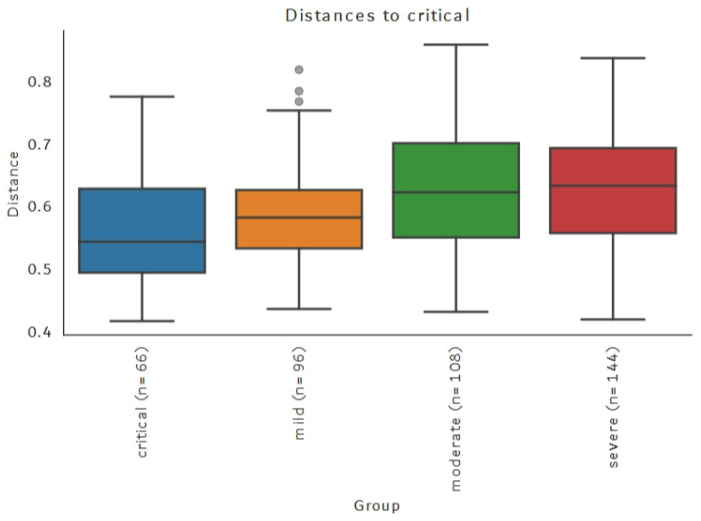
Beta diversity—unweighted UniFrac distances.

Default metrics of beta diversity implemented in QIIME2 were calculated—Jaccard, Bray–Curtis, and weighted and unweighted UniFrac distances. The clustering of samples from critical groups was observed after principal coordinates analysis of the unweighted UniFrac distances matrix (Figure 13 and Figure 14). The clustering was confirmed using the PERMANOVA test statistics pseudo-F with 999 permutations resulting in a q-value of 0.012 for the critical–mild group, 0.018 for the critical–moderate and 0.012 for the critical–severe group. Weighted Unifrac distance also resulted in significant differences between critical–mild and critical–moderate groups (q-value of 0.03). Other metrics used did not result in statistically significant differences in microbial composition based on the groups defined using WHO classification.

### 3.6. Differential Abundance Between Groups Based on Antibiotic Use

When testing the entire population (Mann–Whitney test, *p* = 0.05, FDR) for differential abundance, based on antibiotic use, no significant taxa was found after Benjamini–Hochberg correction, while family Enterococcaceae (*p*-value 0.016856) (Figure S2a,b) and genus Enterococcus (*p*-value 0.016856) (Figure S3a,b) were found significant without Benjamini–Hochberg correction.

When the population was stratified into clinical groups, according to WHO classification, and then tested for antibiotic administration (Kruskal–Wallis test, *p* = 0.05, FDR), it showed genus Coprococcus to be significantly differentially abundant between groups (*p* = 0.0349 after FDR) (Figure S4a,b), being most abundant in the critical group in which all the patients had received antibiotics.

## 4. Discussion

Linking microbiota composition to COVID-19 outcomes, and as a predictor of the severity of the clinical course, has been a challenging and developing field since the onset of the COVID-19 pandemic [22]. For example, now it is well known that SARS-CoV-2 is bacteriophagic, and therefore, to some extent, can directly influence the gut microbiota composition by infecting gut bacteria [7,8]. Furthermore, studies using electron microscopy and isotope labelling indicate that SARS-CoV-2 can replicate in certain bacterial species of the gut microbiota [7,8]. This interaction could also contribute to gut dysbiosis, with reduced microbial diversity and disrupted eubiosis, which are known to be related to impaired inflammation and immune response.

Discovering biological predictors of clinical course severity might prove crucial in identifying individuals with higher risks of fatal outcomes and engaging them in treatment in earlier stages of the disease. In the present study, based on the gut microbiota composition of 41 COVID-19 patients, several biomarkers related to the severity of COVID-19 clinical course have been identified.

Our results showed two genera to be differentially abundant between groups—Halococcus and Coprococcus. Genus Halococcus shows a better correlation with severity of clinical course, with relative abundance increasing with severity of clinical course, being most abundant in the critical group, with relative abundance higher than 10%. Existing studies, linking gut microbiota and severity of COVID-19, did not identify genera Halococcus or identified species Halococcus thailandensis as predictive biomarkers.

The role of the Archaea effect on gut microbiota and health remains poorly studied [23], with sources of intestinal halophilic archaea believed to be salty food [24], which in clinical settings where our samples were collected seems unlikely. The positive health effects of Archaea in the gut microbiota include enhancing energy extraction from food (by the interaction of Methanogenic Archaea with other gut microbes) and degradation of harmful metabolites by Methanogens (potentially reducing disease risks) [25]. As for their clinical relevance, Archaea in gut microbiota are mentioned in the context of being linked to irritable bowel disease (through stimulation of inflammatory cytokine production) [25] and colorectal cancer (altered archaeal communities in gut microbiota are associated with colorectal cancer, but causal links remain unclear) [26]. Generally, increased production of inflammatory cytokines is an important pathophysiological feature of severe COVID-19 clinical course.

More specifically, the genus Halococcus, as salt-tolerant Archaea, is mentioned to contribute to gut microbiota diversity and help reduce hydrogen pressure in the gut, supporting microbial fermentation and contributing to gut homeostasis [27]. As for their clinical relevance, Halococcus, a genus of Haloarchaea, is not well studied in relation to specific diseases but may contribute to microbial dysbiosis and support the growth of pathogenic bacteria through syntrophic interactions [26], such as cross-feeding—a form of syntrophy (a metabolic partnership) where microbes exchange nutrients, which can occur between Halococcus and sulphate-reducing bacteria (SRB) or facultative anaerobes. For example, Halococcus may break down complex compounds into substrates (e.g., hydrogen, acetate) that SRB or Enterobacteriaceae use, fostering their overgrowth [28]. In some individuals, Halococcus and other haloarchaea dominate the gut archaeome, reducing the abundance of beneficial methanogens like Methanobrevibacter smithii, which typically help maintain gut homeostasis by consuming hydrogen, and their suppression can destabilise microbial networks [27]. Dysbiosis driven by Halococcus may exacerbate conditions like inflammatory bowel disease (IBD) or small intestinal bacterial overgrowth (SIBO) through several mechanisms, such as: depleting commensal bacteria that inhibit pathogens, increasing endotoxin production via Gram-negative pathogen expansion and/or disrupting gut-brain axis signalling through altered neurotransmitter precursors (e.g., GABA, serotonin) [29,30].

Although genus Halococcus is the best biomarker of severity of clinical course in our study, the question remains whether it can be used as a predictor of severity at the early stages of the disease.

Genus Coprococcus, which was also identified as differentially abundant between groups, does not show a linear correlation with the severity of the clinical course. Genus Coprococcus was previously identified in the gut microbiota of people infected with COVID-19, with its abundance decreasing in SARS-CoV-2-infected patients [31,32,33,34].

Genus Coprococcus is a key producer of butyrate, a short-chain fatty acid (SCFA) with anti-inflammatory properties, thus helping to maintain intestinal barrier integrity and supporting colonic cell health [35]. It reduces chronic inflammation, potentially lowering cancer risk [35], and is positively correlated with plant-based diets, enhancing SCFA levels, and thus promoting gut health [36]. While, for example, the clinical role of Halococcus is still largely speculative, the genus Coprococcus is showing much clearer disease associations. Increased abundance of Coprococcus has been identified as a potential biomarker of SIBO. On the other hand, its elimination has been linked to clinical improvement in SIBO patients [37]. Certain Coprococcus subgroups have been associated with IBD, obesity and polycystic ovary syndrome (PCOS) [37]. Reduced presence of Coprococcus has been linked to depression, thus emphasising its role in the gut–brain axis [35]. Further studies are needed to clarify these links.

In our dataset, Coprococcus abundance was not linearly correlated with clinical course severity, but was most abundant in the critical group, followed by a moderate group. Some studies claimed a decrease in genera Coprococcus in patients receiving antibiotic treatment [38]. In our study, only seven patients did not receive antibiotic treatment—four of them being in the severe group and three of them in the moderate group.

In an analysis of alpha and beta diversity, critical groups showed significant differences from other groups. For alpha diversity analysis, two tested metrics—Shannon’s and Pielou evenness showed significant differences between critical and other groups. As the observed features index was not significantly different between groups, we can claim that the distribution of taxa in the critical group is more even than in other groups, which is reflected in both Shannon’s and Pielou’s indices, with the highest values observed in the critical group. This observation is in contrast to the majority of studies reporting a decrease in alpha diversity in COVID-19 patients compared to healthy populations [39,40], while studies on infected populations with different severity of the clinical course have inconclusive results. Some studies have shown significantly higher Chao1 and Shannon indices in groups of asymptomatic and severe acute respiratory syndrome coronavirus 2 (SARS-CoV-2) re-positive cases in comparison to groups of convalescents with adverse outcomes [41]. Some report significantly lower Shannon index in patients in the Intensive Care Unit (ICU) compared to patients not in the ICU [42], without classifying them according to the WHO methodology. Other studies show no significant differences in the Shannon index [43] but showed significant differences in Faith’s phylogenetic diversity [44].

It is, of course, impossible to exclude with absolute certainty some external factors potentially affecting the results (for example, presence of contaminant or artefact of sample processing, batch effect, sequencing depth variation or rarefaction method choice).

All reasonable measures of precaution were taken from the very beginning to mitigate these risks. All of the samples were processed under the same conditions. Regarding the batch effect, after sampling, all samples were processed in a single batch (DNA isolation, library preparation and sequencing) and all samples were processed in QIIME2 (following a standard set of analyses published on QIIME’s web—combination of “Atacama soil microbiome” and “Moving pictures” tutorials) as a single operation. We have not observed any bias on the sequencing depth, both of the raw and cleaned data (no significant difference between groups). Rarefaction curves suggested sampling size (based on a sample with the lowest number of reads) was sufficient to represent the overall microbial community. Both raw data from sequencing and after cleaning the data (DADA2) did not show statistically significant differences between groups. Based on all this, we can conclude that, in our case, the potential influence of external factors on the results is highly unlikely.

Due to the often prolonged hospitalisation, numerous factors could influence the gut microbiota composition. These include formulated food, antibiotics and catabolic metabolism, while treated in ICU stay [45].

As for the antibiotic use in our study, all of the patients in the critical group received antibiotic treatment, contrary to the severe group where 4 out of 12 and in a moderate group where 3 out of 9 patients did not receive antibiotics. As antibiotics are one of the most potent disruptors of the gut microbiota, we have also tested differential abundance between patients who were receiving antibiotics and those not receiving them during their stay at the hospital. Therefore, only a small number of patients did not receive antibiotic therapy, reflecting the reliability of the results obtained. When testing the entire population for differential abundance, based on antibiotic use, the family Enterococcaceae and genus Enterococcus were found significant without Benjamini–Hochberg correction. When the population was stratified into clinical groups, according to WHO classification, and then tested for antibiotic administration, it showed genus Coprococcus to be significantly differentially abundant between groups, being most abundant in the critical group in which all the patients had received antibiotics. Based on these results, it is noticeable that neither family Enterococcaceae nor genus Enterococcus were found to be differentially abundant between clinical groups when analysed regardless of antibiotic use. However, genus Coprococcus was found to be differentially abundant between groups, but without linear correlation with severity of the clinical course, when analysing regardless of the antibiotic use. In conclusion, genus Coprococcus was observed to be most abundant in the critical group, in both analytical modalities, which can be logically explained by the fact that all the patients from the critical group had received antibiotic treatment, due to the nature of the clinical course of the disease. On the other hand, some authors mention the decrease in genera Coprococcus in patients receiving antibiotic treatment [38]. This points toward the need for further studies on a larger number of patients, to be able to make more quality conclusions on the possible influence of antibiotic use on the differential abundance findings.

Taking into consideration that the nutrition of patients admitted to the Intensive Care Unit (ICU) is significantly different from patients who were not, in our study 11 out of 12 patients from the critical group were treated in ICU, while only 4 out of 12 severe and 3 out of 9 moderate patients were treated in ICU. None of the eight mild patients was admitted to ICU. Of course, we cannot exclude the possibility that changes in gut microbiota composition related to the severity of clinical course are being co-influenced by the characteristics of medical treatment. Also, it remains unclear whether these changes in gut microbiota composition influenced the severity of COVID-19 clinical course, or vice versa. Therefore, it remains to be elucidated which factors affected the observed increase in both Shannon and Pielou evenness indexes in the critical group, so future studies in this context are also warranted.

The two main strengths of this study are the fact that the less explored relationship between gut microbiota and COVID-19 severity was addressed by identifying Halococcus as a potential microbial marker of disease severity, as well as the fact that we used two different statistical methods—ANCOM and Kruskal–Wallis tests, to validate results of differential abundance, which further strengthens the reliability of our findings.

As for the study limitations, the sample size is relatively small (especially when divided into four clinical groups), which by itself limits the statistical power of the analysis. To address this, future studies on larger datasets are planned. A single fixed time-point study design is not able to capture dynamic changes in gut microbiota composition, which occur with disease progression. However, the main aim of our study was not to follow the dynamic changes in gut microbiome composition with disease progression but to evaluate the possibility of early prediction of critical clinical courses based on the gut microbiome composition in stool samples taken following admission to the hospital. However, we suggest conducting a longitudinal/follow-up study, whenever possible, to determine if the microbiota changes observed are persistent and not transient as the disease progresses. Also, as previously described, many patients, especially in the critical group, received antibiotics as standard therapy, which could have a profound impact on gut microbiota composition, requiring future studies to elucidate this possible effect.

## 5. Conclusions

Our findings indicate the relationship between gut microbiota composition and the clinical course of COVID-19 disease, pointing towards specific distinct features of the group with clinical course marked as critical. Further studies on larger datasets are planned to confirm this.

In a broader sense, our findings might be useful in combating potential future similar pandemics and emerging virus outbreaks. Pointing towards gut microbiota composition markers as a potential tool for early detection of individuals at an increased risk of developing a severe clinical course and altering gut microbiota composition to actively reduce this risk, could be used as potential novel strategies to prevent severe clinical course and fatal outcomes in the future.

## Figures and Tables

**Table 1:** Differentially abundant features identified by both ANCOM and Kruskal–Wallis test, with resulting *p*-values and FDR for Kruskal–Wallis test and W values for ANCOM.

Taxonomic Level	Taxa	Kruskal–Wallis Test		ANCOM
		*p*-Value	FDR	W Value
Phylum	Euryarchaeota	0.0023509	0.021158	16
Class	Halobacteria	0.0023509	0.039965	30
Order	Halobacteriales	0.0023509	0.02586	58
Family	Halococcaceae	0.0023509	0.086984	68

## Data Availability

The data presented in this study are available on request from the corresponding authors, due to legal reasons (sharing of the data requires permission by the Institute for Anthropological Research, Zagreb, Croatia and Dubrava University Hospital, Zagreb, Croatia. Gut microbiome data sequenced in this study are available from EBI’s ENA repository with study accession id: PRJEB81810.

## Supplementary Materials

The following supporting information can be downloaded at: https://www.mdpi.com/article/10.3390/v17040520/s1, Table S1. Relevant symptoms (according to the WHO classification) and duration of illness of the 41 patients. Table S2. Differences between groups of the raw data from sequencing. Table S3. Differences between groups after cleaning the data (DADA2). Figure S1. Differential abundance between groups based on antibiotic use, on a family level, without FDR correction. Figure S2. (a) Differential abundance between groups based on antibiotic use, on a family level, without FDR correction. (b) Differentially abundant family Enterococcaceae between groups based on antibiotic use, without FDR correction. Figure S3. (a) Differential abundance between groups based on antibiotic use, on a genus level, without FDR correction. (b) Differentially abundant genus Enterococcus between groups based on antibiotic use, without FDR correction. Figure S4. (a) Differential abundance between clinical groups based on antibiotic use, on a genus level, with FDR correction. (b) Differentially abundant genus Coprococcus between clinical groups based on anti-biotic use, with FDR correction.

## References

[1] Yang H., Rao Z. (2021). Structural biology of SARS-CoV-2 and implications for therapeutic development. Nat. Rev. Microbiol..

[2] Schirmer M., Smeekens S.P., Vlamakis H., Jaeger M., Oosting M., Franzosa E.A., Ter Horst R., Jansen T., Jacobs L., Bonder M.J. (2016). Linking the Human Gut Microbiome to Inflammatory Cytokine Production Capacity. Cell.

[3] Yeoh Y.K., Zuo T., Lui G.C.-Y., Zhang F., Liu Q., Li A.Y., Chung A.C., Cheung C.P., Tso E.Y., Fung K.S. (2021). Gut microbiota composition reflects disease severity and dysfunctional immune responses in patients with COVID-19. Gut.

[4] Shruti Ahlawat A., Sharma K.K. (2020). Immunological co-ordination between gut and lungs in SARS-CoV-2 infection. Virus Res..

[5] World Health Organisation Coronavirus Disease. https://www.who.int/emergencies/diseases/novel-coronavirus-2019.

[6] Ahmadi Badi S., Tarashi S., Fateh A., Rohani P., Masotti A., Siadat S.D. (2021). From the Role of Microbiota in Gut-Lung Axis to SARS-CoV-2 Pathogenesis. Mediat. Inflamm..

[7] Brogna C., Brogna B., Bisaccia D.R., Lauritano F., Marino G., Montano L., Cristoni S., Prisco M., Piscopo M. (2022). Could SARS-CoV-2 Have Bacteriophage Behavior or Induce the Activity of Other Bacteriophages?. Vaccines.

[8] Brogna C., Costanzo V., Brogna B., Bisaccia D.R., Brogna G., Giuliano M., Montano L., Viduto V., Cristoni S., Fabrowski M. (2023). Analysis of Bacteriophage Behavior of a Human RNA Virus, SARS-CoV-2, through the Integrated Approach of Immunofluorescence Microscopy, Proteomics and D-Amino Acid Quantification. Int. J. Mol. Sci..

[9] Kau A.L., Ahern P.P., Griffin N.W., Goodman A.L., Gordon J.I. (2011). Human nutrition, the gut microbiome and the immune system. Nature.

[10] Luchen C.C., Chibuye M., Spijker R., Simuyandi M., Chisenga C., Bosomprah S., Chilengi R., Schultsz C., Mende D.R., Harris V.C. (2023). Impact of antibiotics on gut microbiome composition and resistome in the first years of life in low- to middle-income countries: A systematic review. PLoS Med..

[11] Ramirez J., Guarner F., Bustos Fernandez L., Maruy A., Sdepanian V.L., Cohen H. (2020). Antibiotics as Major Disruptors of Gut Microbiota. Front. Cell. Infect. Microbiol..

[12] McDonald D., Ackermann G., Khailova L., Baird C., Heyland D., Kozar R., Lemieux M., Derenski K., King J., Vis-Kampen C. (2016). Extreme Dysbiosis of the Microbiome in Critical Illness. mSphere.

[13] Beam A., Clinger E., Hao L. (2021). Effect of Diet and Dietary Components on the Composition of the Gut Microbiota. Nutrients.

[14] World Health Organization Living Guidance for Clinical Management of COVID-19. https://iris.who.int/bitstream/handle/10665/349321/WHO-2019-nCoV-clinical-2021.2-eng.pdf.

[15] Bolyen E., Rideout J.R., Dillon M.R., Bokulich N.A., Abnet C.C., Al-Ghalith G.A., Alexander H., Alm E.J., Arumugam M., Asnicar F. (2019). Reproducible, interactive, scalable and extensible microbiome data science using QIIME 2. Nat. Biotechnol..

[16] Callahan B.J., McMurdie P.J., Rosen M.J., Han A.W., Johnson A.J.A., Holmes S.P. (2016). DADA2: High-resolution sample inference from Illumina amplicon data. Nat. Methods.

[17] Katoh K., Misawa K., Kuma K., Miyata T. (2002). MAFFT: A novel method for rapid multiple sequence alignment based on fast Fourier transform. Nucleic Acids Res..

[18] Price M.N., Dehal P.S., Arkin A.P. (2010). FastTree 2–approximately maximum-likelihood trees for large alignments. PLoS ONE.

[19] Bokulich N.A., Kaehler B.D., Rideout J.R., Dillon M., Bolyen E., Knight R., Huttley G.A., Gregory Caporaso J. (2018). Optimizing taxonomic classification of marker-gene amplicon sequences with QIIME 2’s q2-feature-classifier plugin. Microbiome.

[20] Molano L.A.G., Vega-Abellaneda S., Manichanh C. (2024). GSR-DB: A manually curated and optimized taxonomical database for 16S rRNA amplicon analysis. mSystems.

[21] Mandal S., Van Treuren W., White R.A., Eggesbø M., Knight R., Peddada S.D. (2015). Analysis of composition of microbiomes: A novel method for studying microbial composition. Microb. Ecol. Health Dis..

[22] Zhou J., Yang X., Yang Y., Wei Y., Lu D., Xie Y., Liang H., Cui P., Ye L., Huang J. (2023). Human microbiota dysbiosis after SARS-CoV-2 infection have the potential to predict disease prognosis. BMC Infect. Dis..

[23] Herrera G., Paredes-Sabja D., Patarroyo M.A., Ramírez J.D., Muñoz M. (2021). Updating changes in human gut microbial communities associated with *Clostridioides difficile* infection. Gut Microbes.

[24] Demonfort Nkamga V., Henrissat B., Drancourt M. (2017). Archaea: Essential inhabitants of the human digestive microbiota. Hum. Microbiome J..

[25] Gaci N., Borrel G., Tottey W., O’Toole P.W., Brugère J.F. (2014). Archaea and the human gut: New beginning of an old story. World J. Gastroenterol..

[26] Duller S., Moissl-Eichinger C. (2024). Archaea in the Human Microbiome and Potential Effects on Human Infectious Disease. Emerg. Infect. Dis..

[27] Kim J.Y., Whon T.W., Lim M.Y., Kim Y.B., Kim N., Kwon M.S., Kim J., Lee S.H., Choi H.J., Nam I.H. (2020). The human gut archaeome: Identification of diverse haloarchaea in Korean subjects. Microbiome.

[28] Kouzuma A., Kato S., Watanabe K. (2015). Microbial interspecies interactions: Recent findings in syntrophic consortia. Front. Microbiol..

[29] DeGruttola A.K., Low D., Mizoguchi A., Mizoguchi E. (2016). Current Understanding of Dysbiosis in Disease in Human and Animal Models. Inflamm. Bowel Dis..

[30] Zhang P., Kong L., Huang H., Pan Y., Zhang D., Jiang J., Shen Y., Xi C., Lai J., Ng C.H. (2022). Gut Microbiota—A Potential Contributor in the Pathogenesis of Bipolar Disorder. Front. Neurosci..

[31] Tao W., Zhang G., Wang X., Guo M., Zeng W., Xu Z. (2020). Analysis of the intestinal microbiota in COVID-19 patients and its correlation with the inflammatory factor IL-18. Med. Microecol..

[32] Wang M., Zhang Y., Li C., Chang W., Zhang L. (2023). The relationship between gut microbiota and COVID-19 progression: New insights into immunopathogenesis and treatment. Front. Immunol..

[33] Li S., Yang S., Zhou Y., Disoma C., Dong Z., Du A., Zhang Y., Chen Y., Huang W., Chen J. (2021). Microbiome Profiling Using Shotgun Metagenomic Sequencing Identified Unique Microorganisms in COVID-19 Patients with Altered Gut Microbiota. Front. Microbiol..

[34] Wu Y., Cheng X., Jiang G., Tang H., Ming S., Tang L., Lu J., Guo C., Shan H., Huang X. (2021). Author Correction: Altered oral and gut microbiota and its association with SARS-CoV-2 viral load in COVID-19 patients during hospitalization. npj Biofilms Microbiomes.

[35] Notting F., Pirovano W., Sybesma W., Kort R. (2023). The butyrate-producing and spore-forming bacterial genus Coprococcus as a potential biomarker for neurological disorders. Gut Microbiome.

[36] Tomova A., Bukovsky I., Rembert E., Yonas W., Alwarith J., Barnard N.D., Kahleova H. (2019). The Effects of Vegetarian and Vegan Diets on Gut Microbiota. Front. Nutr..

[37] Guo H., Chen Y., Dong W., Lu S., Du Y., Duan L. (2024). Fecal Coprococcus, hidden behind abdominal symptoms in patients with small intestinal bacterial overgrowth. J. Transl. Med..

[38] Cao J., Wang C., Zhang Y., Lei G., Xu K., Zhao N., Lu J., Meng F., Yu L., Yan J. (2021). Integrated gut virome and bacteriome dynamics in COVID-19 patients. Gut Microbes.

[39] Galperine T., Choi Y., Pagani J.L., Kritikos A., Papadimitriou-Olivgeris M., Méan M., Scherz V., Opota O., Greub G., Guery B. (2023). Temporal changes in fecal microbiota of patients infected with COVID-19: A longitudinal cohort. BMC Infect. Dis..

[40] Righi E., Vecchia I.D., Auerbach N., Morra M., Górska A., Sciammarella C., Lambertenghi L., Gentilotti E., Mirandola M., Tacconelli E. (2024). Gut Microbiome Disruption Following SARS-CoV-2: A Review. Microorganisms.

[41] Lin R., Xiao M., Cao S., Sun Y., Zhao L., Mao X., Chen P., Tong X., Ou Z., Zhu H. (2022). Distinct gut microbiota and health outcomes in asymptomatic infection, viral nucleic acid test re-positive, and convalescent COVID-19 cases. mLife.

[42] Shen Y., Yu F., Zhang D., Zou Q., Xie M., Chen X., Yuan L., Lou B., Xie G., Wang R. (2022). Dynamic Alterations in the Respiratory Tract Microbiota of Patients with COVID-19 and its Association with Microbiota in the Gut. Adv. Sci..

[43] Mazzarelli A., Giancola M.L., Fontana A., Piselli P., Binda E., Trivieri N., Mencarelli G., Marchioni L., Vulcano A., De Giuli C. (2022). Gut microbiota composition in COVID-19 hospitalized patients with mild or severe symptoms. Front. Microbiol..

[44] Fabbrini M., D’amico F., van der Gun B.T.F., Barone M., Conti G., Roggiani S., Wold K.I., Vincenti-Gonzalez M.F., de Boer G.C., Veloo A.C.M. (2024). The gut microbiota as an early predictor of COVID-19 severity. mSphere.

[45] Gu S., Chen Y., Wu Z., Chen Y., Gao H., Lv L., Guo F., Zhang X., Luo R., Huang C. (2020). Alterations of the Gut Microbiota in Patients with Coronavirus Disease 2019 or H1N1 Influenza. Clin. Infect. Dis..

