# Supplementary material for: Relationship Between Gut Microbiota and the Clinical Course of COVID-19 Disease
Source: Viruses. 2025 Apr 2;17(4):520. doi: 10.3390/v17040520 (PMC12031135; doi:10.3390/v17040520)
Supplement: Supplementary file 1 [file viruses-17-00520-s001.zip › viruses-3267970-supplementary.pdf]

viruses

MDPI

Article

Relationship between Gut Microbiota and the Clinical Course of COVID-19 Disease

Supplementary data:

Table S1. Relevant symptoms (according to the WHO classification) and duration of illness of the 41 patients.

Patient number	Category (according to WHO)	Duration of illness (days)	Mild	Moderate	Severe	Critical
			No viral pneumonia or hypoxia	Pneumonia SpO2 ≥ 90% on room air	Severe pneumonia<br>(Fever<br>Cough<br>Dyspnoea)<br>and at least one of the following:<br>Respiratory rate > 30 breaths/min<br>Severe respiratory distress<br>SpO2 < 90% on room air	Worsening of respiratory symptoms<br>ARDS<br>Sepsis<br>Septic shock<br>Acute thrombosis<br>Death
01	mild	20	x			
02	moderate	12		x		
03	mild	30	x			
04	moderate	15		x		
05	moderate	20		x		
06	mild	22	x			
07	severe	21			Severe pneumonia<br>SpO2 < 90% on room air	
08	mild	15	x			
09	mild	25	x			
10	mild	41	x			
11	moderate	30		x		
12	severe	50			Severe pneumonia<br>SpO2 < 90% on room air	
13	severe	51			Severe pneumonia<br>Severe respiratory distress<br>SpO2 < 90% on room air	
14	moderate	22		x		
15	mild	64	x			

16	severe	51			Severe pneumonia<br>SpO2 < 90% on room air	
17	severe	29			Severe pneumonia<br>Respiratory rate > 30 breaths/min<br>SpO2 < 90% on room air	
18	moderate	53		x		
19	severe	77			Severe pneumonia<br>SpO2 < 90% on room air	
20	severe	37			Severe pneumonia<br>Severe respiratory distress<br>SpO2 < 90% on room air	
21	severe	25			Severe pneumonia<br>Severe respiratory distress<br>SpO2 < 90% on room air	
22	moderate	47		x		
23	critical	47				Worsening of respiratory symptoms<br>Septic shock<br>Death
24	critical	20				Worsening of respiratory symptoms<br>Septic shock<br>Death
25	critical	8				Worsening of respiratory symptoms<br>ARDS<br>Death
26	critical	80				Worsening of respiratory symptoms<br>Sepsis<br>Death
27	critical	20				Worsening of respiratory symptoms<br>ARDS<br>Death
28	critical	39				Worsening of respiratory symptoms<br>Sepsis<br>Death
29	critical	65				Worsening of respiratory symptoms

						Sepsis<br>Death
30	mild	54	x			
31	severe	41			Severe pneumonia<br>SpO2 < 90% on room air	
32	moderate	33		x		
33	severe	38			Severe pneumonia<br>SpO2 < 90% on room air	
34	moderate	10		x		
35	critical	57				Worsening of respiratory symptoms<br>ARDS<br>Sepsis<br>Death
36	critical	37				Worsening of respiratory symptoms<br>ARDS<br>Death
37	severe	18			Severe pneumonia<br>Severe respiratory distress<br>SpO2 < 90% on room air	
38	critical	14				Worsening of respiratory symptoms<br>ARDS<br>Death
39	severe	19			Severe pneumonia<br>Respiratory rate > 30 breaths/min<br>SpO2 < 90% on room air	
40	critical	25				Worsening of respiratory symptoms<br>Sepsis<br>Death
41	critical	10				Worsening of respiratory symptoms<br>Sepsis<br>Death

Table S2. Differences between groups of the raw data from sequencing.

	mean	std. dev.
mild	94731.25	15312.98
moderate	98080	18048.51
severe	94666.67	19442.36
critical	87773.08	16176.67
Test Statistic F:	0.6673	
p-value	0.577516.	

Table S3. Differences between groups after cleaning the data (DADA2).

	mean	st.dev.
mild	51152.63	10500.59
moderate	51843.44	9543.503
severe	48605.75	10846.18
critical	50931.67	9118.35
Test Statistic F:	0.422	
p-value	0.73832	

Feature ID #q2:types	Taxon categorical	Confidence categorical
8b4280f0e24a163c61a5e7f31e9b8922	k__Archaea; p__Euryarchaeota; c__Halobacteria	0.8442767182807873
12d4d8d1aab930d5d821d15c46d14f21	k__Archaea; p__Euryarchaeota; c__Halobacteria; o__Halobacteriales; f__Halococcaceae; g__Halococcus; s__Halococcus_thailandensis	0.8594735550341441
3f01ad94e9f41132ded7e0c322bd43f9	k__Archaea; p__Euryarchaeota; c__Halobacteria; o__Halobacteriales; f__Halococcaceae; g__Halococcus; s__Halococcus_thailandensis	0.8702761613098806
4d2ebfdf2016618355c11d587d0357e3	k__Archaea; p__Euryarchaeota; c__Halobacteria; o__Halobacteriales; f__Halococcaceae; g__Halococcus; s__Halococcus_thailandensis	0.8383270851922812
76cee239197687206fbb0eb083081ce2	k__Archaea; p__Euryarchaeota; c__Halobacteria; o__Halobacteriales; f__Halococcaceae; g__Halococcus; s__Halococcus_thailandensis	0.7915479268421094
b610de8a5e1cf52bf2bba46063918137	k__Archaea; p__Euryarchaeota; c__Halobacteria; o__Halobacteriales; f__Halococcaceae; g__Halococcus; s__Halococcus_thailandensis	0.8556971359043517
ba1e509f03451781834d4bee037e2703	k__Archaea; p__Euryarchaeota; c__Halobacteria; o__Halobacteriales; f__Halococcaceae; g__Halococcus; s__Halococcus_thailandensis	0.8170071318848503
edb5bb60e5c3bf37985c76847063c32c	k__Archaea; p__Euryarchaeota; c__Halobacteria; o__Halobacteriales; f__Halococcaceae; g__Halococcus; s__Halococcus_thailandensis	0.8394061864300322

Figure S1. Differential abundance between groups based on antibiotic use, on a family level, without FDR correction.

The table below shows at most 500 features ranked by their p values, with significant features highlighted in orange.

Name	Pvalues	FDR	Statistics	logCPM	View
Enterococcaceae	0.016856	0.64052	51.0	19.0263	
Staphylococcaceae	0.066254	0.72972	66.0	13.164	
Odoribacteraceae	0.093164	0.72972	166.5	13.0422	

Figure S2a. Differential abundance between groups based on antibiotic use, on a family level, without FDR correction.

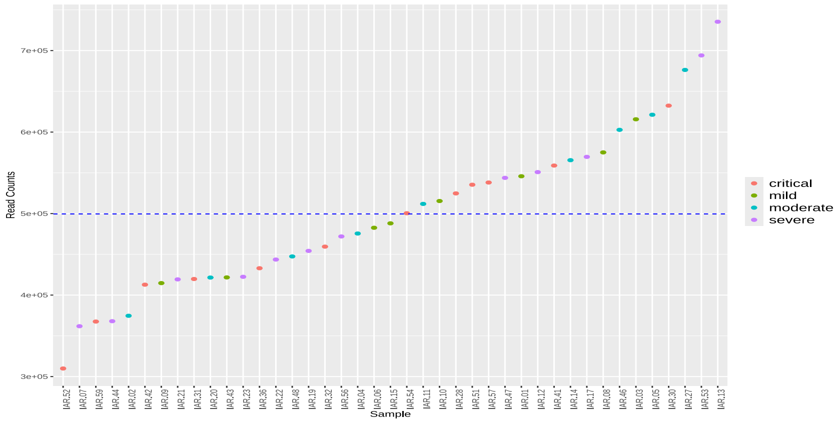

Figure S2b. Differentially abundant family Enterococcaceae between groups based on antibiotic use, without FDR correction.

The table below shows at most 500 features ranked by their p values, with significant features highlighted in orange.

Name	Pvalues	FDR	Statistics	logCPM	View
Enterococcus	0.016856	0.71661	51.0	19.2451	
Odoribacter	0.042586	0.71661	175.5	12.9953	
Negativibacillus	0.058974	0.71661	73.5	7.27133	

Figure S3a. Differential abundance between groups based on antibiotic use, on a genus level, without FDR correction.

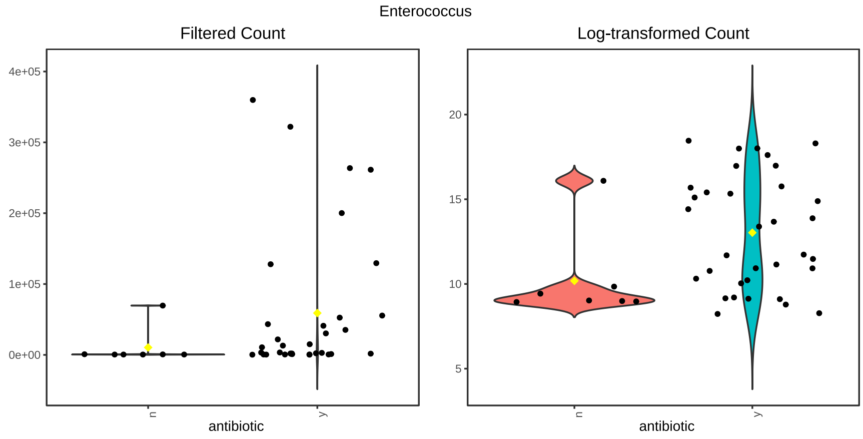

Figure S3b. Differentially abundant genus Enterococcus between groups based on antibiotic use, without FDR correction.

The table below shows at most 500 features ranked by their p values, with significant features highlighted in orange.

Name	Pvalues	FDR	Statistics	logCPM	View
Coprococcus	4.2078E-4	0.034925	22.499	11.6612	
Pygmaiobacter	0.0047913	0.15969	16.851	9.8991	
Halococcus	0.0060521	0.15969	16.294	15.9059	

Figure S4a. Differential abundance between clinical groups based on antibiotic use, on a genus level, with FDR correction.

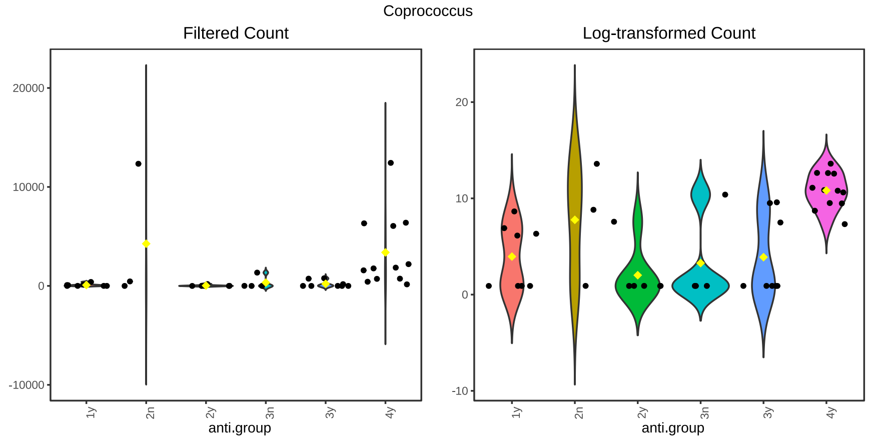

Figure S4b. Differentially abundant genus Coprococcus between clinical groups based on antibiotic use, with FDR correction.
